# Nitrogen and Carbon Status Are Integrated at the Transcriptional Level by the Nitrogen Regulator NtrC *In Vivo*

**DOI:** 10.1128/mBio.00881-13

**Published:** 2013-11-19

**Authors:** Jörg Schumacher, Volker Behrends, Zhensheng Pan, Dan R. Brown, Franziska Heydenreich, Matthew R. Lewis, Mark H. Bennett, Banafsheh Razzaghi, Michal Komorowski, Mauricio Barahona, Michael P. H. Stumpf, Sivaramesh Wigneshweraraj, Jacob G. Bundy, Martin Buck

**Affiliations:** Division of Cell & Molecular Biology, Imperial College London, London, United Kingdom^a^; Department of Surgery and Cancer, Faculty of Medicine, Imperial College London, London, United Kingdom^b^; MRC Centre for Molecular Bacteriology and Infection, Imperial College London, London, United Kingdom^c^; Division of Molecular Biosciences, Imperial College London, London, United Kingdom^d^; Department of Mathematics, Imperial College London, London, United Kingdom^e^; Institute of Fundamental Technological Research, Polish Academy of Sciences, Warsaw, Poland^f^

## Abstract

Nitrogen regulation in *Escherichia coli* is a model system for gene regulation in bacteria. Growth on glutamine as a sole nitrogen source is assumed to be nitrogen limiting, inferred from slow growth and strong NtrB/NtrC-dependent gene activation. However, we show that under these conditions, the intracellular glutamine concentration is not limiting but 5.6-fold higher than in ammonium-replete conditions; in addition, α-ketoglutarate concentrations are elevated. We address this glutamine paradox from a systems perspective. We show that the dominant role of NtrC is to regulate *glnA* transcription and its own expression, indicating that the glutamine paradox is not due to NtrC-independent gene regulation. The absolute intracellular NtrC and GS concentrations reveal molecular control parameters, where NtrC-specific activities were highest in nitrogen-starved cells, while under glutamine growth, NtrC showed intermediate specific activity. We propose an *in vivo* model in which α-ketoglutarate can derepress nitrogen regulation despite nitrogen sufficiency.

## IMPORTANCE

Nitrogen is the most important nutrient for cell growth after carbon, and its metabolism is coordinated at the metabolic, transcriptional, and protein levels. We show that growth on glutamine as a sole nitrogen source, commonly assumed to be nitrogen limiting and used as such as a model system for nitrogen limitation, is in fact nitrogen replete. Our integrative quantitative analysis of key molecules involved in nitrogen assimilation and regulation reveal that glutamine is not necessarily the dominant molecule signaling nitrogen sufficiency and that α-ketoglutarate may play a more important role in signaling nitrogen status. NtrB/NtrC integrates α-ketoglutarate and glutamine signaling—sensed by the UTase (*glnD*) and PII (*glnB*), respectively—and regulates the nitrogen response through self-regulated expression and phosphorylation-dependent activation of the nitrogen (*ntr*) regulon. Our findings support α-ketoglutarate acting as a global regulatory metabolite.

## INTRODUCTION

Integrative systems biology approaches provide comprehensive data on the physiological state of the cell that can reveal control parameters and limits of regulatory networks, inform predictive models, and guide metabolic engineering approaches. Ultimately, they can be used to understand adaptive processes. Nitrogen (N) is a major nutrient for cells, and nitrogen regulation and metabolism have been extensively studied (1).

The central nitrogen metabolic circuit is conserved in the vast majority of plants, archaea, and bacteria. Ammonium is the preferred nitrogen source of most bacteria, and nitrogen assimilation involves the enzymes glutamate dehydrogenase (GDH), glutamine synthetase (GS), and glutamate synthase (or glutamate-oxoglutarate amidotransferase [GOGAT]). Glutamate provides 88% of nitrogen for the synthesis of all nitrogen-containing cellular compounds (1) but also serves as a major compound to maintain the K^+^ pool (2). GDH reversibly aminates α-ketoglutarate (α-KG) to glutamate but has a higher *K*_*m*_ for ammonium (>1 mM) than GS (*K*_*m*_ < 200 µM) and appears to play a role in nitrogen assimilation only under energy- and carbon (C)-limiting conditions (2). GS aminates glutamate to glutamine, the amine donor for the remaining 12% of nitrogen-containing compounds. During growth in saturating glucose conditions, glutamate is nearly exclusively produced through amidotransfer from glutamine to α-KG by GOGAT (3), so that nitrogen assimilation from ammonium into glutamine and glutamate would rely essentially on GS. GS activity is posttranslationally regulated by adenylylation in response to the glutamine and α-KG levels (4). Nitrogen assimilation into glutamine is regulated primarily through expression and posttranslational modification of GS.

Glutamine is thought to be the main intracellular signal for nitrogen availability in *Escherichia coli* and most other bacteria. Its levels are sensed by the uridyltransferase/uridylyl-removing enzyme (UT/UR), which sits at the top of a well-studied regulatory cascade involving several proteins (5). At low glutamine levels, UT/UR uridylylates the paralogous transducers/regulators PII and GlnK, which are thought to have some redundant functions. PII-UMP stimulates the adenylate-removing activity of the bifunctional adenylylate transferase/removing enzyme (AT/AR) to increase the catalytic activity of GS. The nonuridylylated PII activates the adenyl transferase activity of GS. PII also controls the activity of the bifunctional enzyme NtrB, reducing its histidine kinase and stimulating its regulated phosphatase activity (6). These PII activities were shown to be modulated *in vitro* through direct binding of α-KG, ATP, and ADP, presumably to coordinate carbon and energy with nitrogen assimilation (7). The NtrB/NtrC two-component system (also called NRII and NRI) regulates transcription of the *ntr* regulon, comprising 27 operons involved in nitrogen scavenging, metabolism, and regulation (8). NtrC is a bacterial enhancer binding protein (bEBP) that activates the alternative σ^54^ RNA polymerase (9, 10). NtrB phosphorylates the NtrC receiver domain in response to nitrogen status, triggering conformational changes that favor formation of higher order NtrC oligomers, which is thought to be a prerequisite for transcription activation (11). However, NtrC can also act as a transcriptional repressor, and the role of its phosphorylation at repressed promoters is unclear. One consequence of this alternative regulatory mechanism is the strict requirement of an enhancer binding protein for the activation of σ^54^-dependent transcription (12).

The *glnALG* operon is central to nitrogen metabolism. It codes for GS, NtrB, and NtrC and comprises three promoters (*glnAp1*, *glnAp2*, and *glnLp*) with potentially three NtrC-dependent autofeedback loops (13). The cAMP receptor protein (CRP) also regulates transcription of *glnAp1* and *glnAp2* in response to C source availability (14).

Many genetic studies on the regulation of N assimilation have used glutamine as the sole N source (e.g., references 2 and 15–19). It was proposed that active import of glutamine by the high-affinity glutamine transporter (*glnHPQ*) is too slow to support fast growth and the necessary metabolic fluxes from glutamine, and so would account for intracellular N-limiting conditions that result in the strong NtrC-dependent upregulation of the *ntr* regulon (20). Ammonium is the preferred N source, allowing fast growth and resulting in the repression of the *ntr* regulon and the transport of glutamine by the glutamine transporter (*glnHPQ*) (21). More generally, slow growth of enteric bacteria such as *Salmonella* in a number of nonoptimal nitrogen sources, such as arginine and proline, in conjunction with strong NtrC-dependent transcription, has been defined as nitrogen limiting due to low intracellular glutamine concentrations (22).

Here, we report transcript, protein, and metabolite levels of the key molecules (transcripts, proteins, metabolites) involved in N regulation and metabolism in wild-type *Escherichia coli* and an isogenic *glnG* deletion, grown in batch cultures under N-rich (10 mM NH_4_Cl), glutamine (5 mM glutamine), and N-starved (3 mM NH_4_Cl) conditions. We reasoned that direct measurements of key intracellular molecule levels would (i) provide a more quantitative description of the N-adaptive physiological states and how these relate to transcription control, (ii) not suffer from potential pleiotropic effects in gene deletion studies, and (iii) provide multilevel control parameters of the N regulatory network. Strikingly, we found that intracellular glutamine levels under glutamine conditions were nearly 6-fold higher than for growth under nitrogen-rich conditions, which is inconsistent with glutamine being a sufficient and dominant signal of nitrogen status regulating NtrC-dependent transcription. We propose a modified regulatory network with a predominant role for α-ketoglutarate.

## RESULTS

### NtrC is required for optimal growth in both nitrogen*-*poor and -rich media.

We used wild-type (WT) *E. coli* strain NCM3722 and nitrogen regimes that allow comparisons with many previous studies of the Ntr system (e.g., see references 8, 23, 24, and 25). NCM3722 is prototrophic and a close reconstruction of the originally sequenced but genetically corrupted MG1655 *E. coli* K-12 strain, which suffers from various growth defects (23). We produced the NCM3722Δ*glnG* (Δ*glnG*) strain, which lacks the coding sequence for NtrC, through phage transduction from donor strain JW3841-1 (BW25113Δ*glnG*), provided by the Keio collection.

We directly quantified key metabolites, transcripts, and proteins under three different nitrogen regimes: two steady states and one transient physiological state. We used glucose as the carbon and energy sources throughout, in a defined minimal medium. Steady-state samples were taken during exponential growth (optical density at 600 nm [OD_600_] between 0.4 and 0.6). The initial nitrogen contents were 10 mM NH_4_Cl, 5 mM glutamine, and 3 mM NH_4_Cl. Ammonium is the preferred nitrogen source for most bacteria, and 10 mM NH_4_Cl is nitrogen replete (NH_4_ rich). Growth on glutamine as the sole nitrogen source (glutamine) is slow, and NtrC-regulated genes are strongly upregulated during glutamine-dependent growth. Hence, growth on glutamine has been extensively used in earlier studies as a model of nitrogen limitation. To capture changes in the physiological state of cells as they pass from nitrogen-replete to nitrogen-poor growth conditions, we carried out ammonium run-out experiments (nitrogen starved) from an initial 3 mM NH_4_Cl.

**FIG 1:**
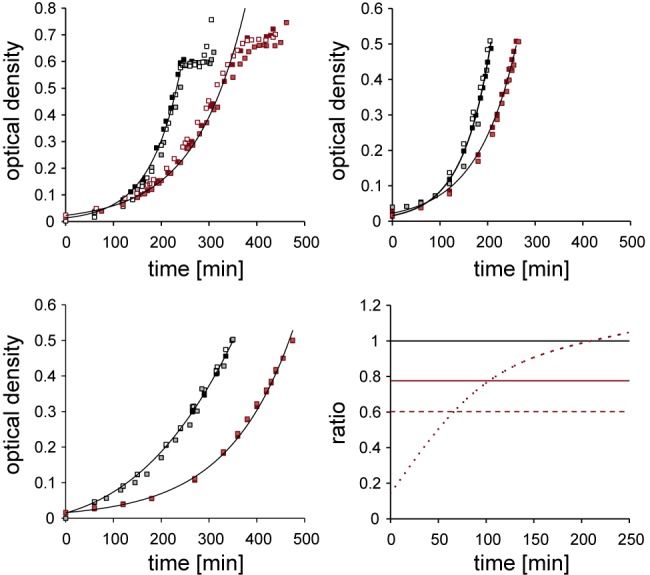
Growth curves of *E. coli* NCM3722 (black) and NCM3722Δ*glnG* (red) strains under different nitrogen regimes. Samples for targeted quantitative determination of metabolites, transcripts, and proteins *in vivo* were from these cultures during exponential growth in ammonium-rich conditions (top left), in glutamine (top right), or 10 min after growth arrest, indicating nitrogen-starved conditions (bottom left). Growth rates for ammonium-starved conditions (Table 1) were derived from logarithmic growth before nitrogen run out. Bottom right, growth rate differences (ΔOD_600_/Δ*t*) between NCM3722 and NCM3722Δ*glnG* strains under different regimes: ammonium rich (long dash), glutamine (short dash), ammonium starved (solid).

As expected, growth of WT and Δ*glnG* strains in NH_4_Cl was considerably faster than growth in glutamine (Fig. 1). We observed that following a period of balanced exponential growth in 3 mM NH_4_Cl, doublings slowed abruptly in WT and Δ*glnG* strains. For nitrogen-starved conditions, samples were taken 10 min after growth stopped (Fig. 1C).

The doubling time of NCM3722 in defined nitrogen-rich minimal medium was 42 min, which is identical with reported doubling times of wild-type *E. coli* (26) and very similar to those reported in early studies with wild-type *E. coli* (e.g., see reference 27) (Table 1). Significant transcriptomic, metabolomic, and proteomic differences have been reported between closely related *E. coli* strains grown under identical conditions, underlining the importance of using isogenic strains in comparative systems biology studies (28, 29). Our results support the use of NCM3722 as a reference strain for wild-type *E. coli* (23).

**TABLE 1:** Doubling times (*g*) and growth rates (μ) of wild-type NCM3722 and NCM3722Δ*glnG* strains under different growth regimes^*a*^

Genotype	N status	*g* (min) ± SE	μ (h^−1^) ± SE
WT	NH_4_ rich	42.1 ± 0.9	0.99 ± 0.02
WT	Glutamine	75.24 ± 0	0.55 ± 0
WT	NH_4_ starved	45.5 ± 3.2	1.04 ± 0.08
Δ*glnG*	NH_4_ rich	53.8 ± 0.96	0.77 ± 0.01
Δ*glnG*	Glutamine	95.7 ± 1.7	0.14 ± 0.01
Δ*glnG*	NH_4_ starved	74 ± 2.1	0.56 ± 0.02

^a^ Growth rates for ammonium-starved cells are derived from logarithmic growth before ammonium run-out (see Fig. 1). SE = 1 standard error of the mean.

For all conditions tested, the Δ*glnG* strain grew significantly slower than the WT. We conclude that NtrC confers fitness under nitrogen-rich conditions, in addition to its well-established role in adaptation to nitrogen starvation (8). The statistically indistinguishable growth rates of the WT in high- and low-ammonium media prior to run-out imply that ammonium uptake under those conditions is not limiting, consistent with ammonia crossing the bacterial membrane at neutral pH and supporting optimal growth. The NtrC-regulated high-affinity ammonium transporter AmtB is activated only at very low (below 30 µM) ammonium concentrations, since passive transport is sufficient at higher concentrations (30). Therefore, the growth phenotypes in Fig. 1 should not be a simple consequence of insufficient ammonium uptake. To test this, we measured ammonium depletion in the media during exponential growth under high and low initial ammonium concentrations (see Fig. S1 in the supplemental material). Ammonium consumption strictly correlated with growth, and ammonium uptake was indistinguishable between high- and low-ammonium conditions during the initial exponential growth phase, consistent with no dependence on AmtB. The early onset of slowed growth of the Δ*glnG* strain under both ammonium conditions is therefore not easily explained by a lack of intracellular ammonium.

### The glutamine paradox.

Growth in glutamine was markedly slower than in media containing ammonium, for both the WT and Δ*glnG* strains. Unlike ammonia, glutamine cannot freely diffuse across the membrane and is thought to require import by the glutamine transporter encoded by the NtrC-regulated *glnHPQ* operon. To see if intracellular glutamine was limiting due to slow transport, we measured the intracellular concentrations of glutamine by liquid chromatography-mass spectrometry (LC/MS), after confirming that our sampling approach allowed accurate, rapid, and separate quantification of metabolites present in both the exo- and endometabolomes (see Fig. S2 in the supplemental material).

**FIG 2:**
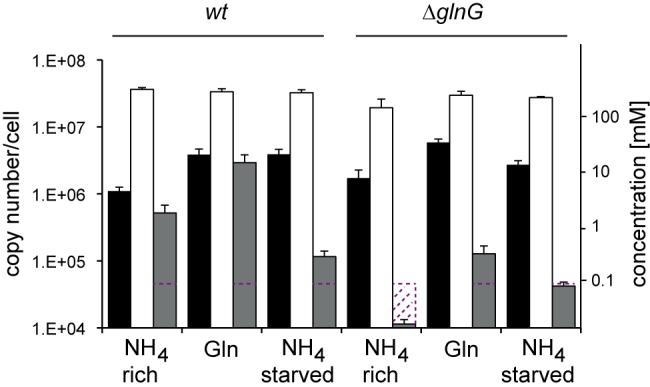
Intracellular concentrations (molecules/cell) of α-ketoglutarate (black), glutamine (white), and glutamine (gray) under different nitrogen regimes as indicated. Note logarithmic scale on the *y* axes. Red dotted line indicates quantification limit. Error bars indicate one standard error of the mean across minimally three biological replicates.

Intracellular glutamine concentrations were high under glutamine growth (Fig. 2). This calls into question the reported role of intracellular glutamine as a sufficient and dominant signal for nitrogen status, and so we quantified two other key metabolites in nitrogen assimilation, glutamate and α-KG. All samples had similar intracellular glutamate concentrations (Fig. 2), supporting the importance and homeostatic protection of the glutamate pool in enteric bacteria (2), but intracellular α-KG accumulated in nitrogen-starved and glutamine conditions. Interestingly, we also found α-KG in the supernatants of glutamine and nitrogen-starved cultures (data not shown), indicating active export of α-KG to prevent its hyperaccumulation in the cells. We conclude that the key metabolite concentrations of the nitrogen assimilation pathway (glutamine in particular) are not limited under glutamine growth and therefore cannot account for the slow-growth phenotype or the transcriptional activation of NtrC-dependent genes.

This apparent paradox calls for an alternative hypothesis to explain the glutamine phenotypes. To better understand the interplay between physiological state and nitrogen regulation by NtrC, we measured the α-KG, glutamate, and glutamine concentrations in the Δ*glnG* strain. The glutamine concentrations in the Δ*glnG* strain were at least 10-fold lower than those of the WT under nitrogen-rich and glutamine growth conditions and still measurably lower under starved conditions, although exact measurements of fold change were not possible, as levels were below the limit of quantification for nitrogen-rich and nitrogen-starved samples (Fig. 2). The slower growth in the Δ*glnG* strain under nitrogen-rich conditions may be a direct consequence of lacking glutamine. However, the relatively high glutamine concentration of the Δ*glnG* strain under glutamine growth suggests that glutamine limitation does not exclusively account for the severe growth phenotype under these conditions. During the course of the experiments under glutamine but not under ammonium growth, growth rate differentials between the WT and Δ*glnG* strains (Fig. 1D) indicate that relative growth rates of the Δ*glnG* strain partly recover compared to those of the WT, suggesting a potential role for NtrC during adaptation to glutamine growth. To better understand the role of NtrC in N adaptation, we turned our attention to the NtrC regulon and its distinct roles in the growth phenotypes, since metabolite concentrations of the central N pathway are not limiting (Fig. 2).

### Revisiting the regulation of NtrC*-*dependent genes.

**FIG 3:**
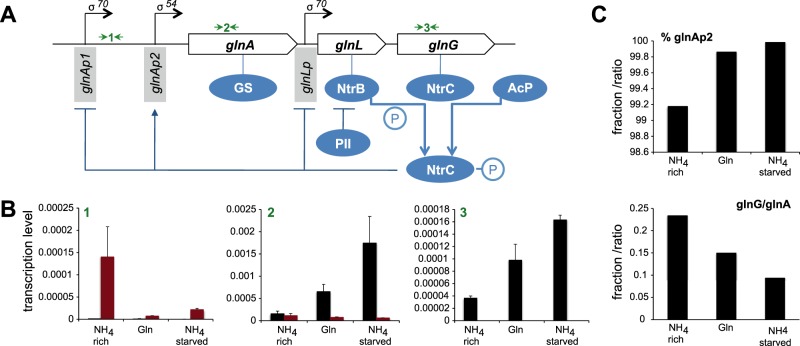
Transcription from different NtrC-dependent promoters within the *glnALG* operon under different nitrogen regimes. (A) Scheme of the *glnALG* operon architecture and NtrC-mediated feedback loops acting at σ^70^- and σ^54^-dependent promoters. Black arrows indicate three primer pairs used for quantitative PCR of *glnAp1* (1), *glnA* (2), and *glnG* (3). (B) mRNA levels relative to 16 s mRNA of *E. coli* NCM3722 (black) and NCM3722Δ*glnG* (red) strains comprising *glnAp1* (1), *glnA* (2), and *glnG* (3) message. Transcript levels between *E. coli* NCM3722 and NCM3722Δ*glnG* strains confirm the regulatory roles of NtrC at those promoters. (C) The percentage of *glnAp2* activity of total *glnA* transcription was derived from *glnA* transcripts minus *glnAp1* transcripts. The ratio of *glnG* transcripts relative to *glnA* transcripts reflects on the regulation at the *glnLp* promoter.

Regulated NtrC expression involves two negative and one positive autoregulatory feedback mechanisms through the *glnAp1*, *glnLp*, and *glnAp2* promoters, to which NtrC binds within the *glnALG* operon (Fig. 3A). *glnAp2* comprises two high-affinity enhancer NtrC binding sites (1 and 2) and two low-affinity NtrC binding sites further downstream (3 and 4), proposed to act as governor sites to limit *glnA* transcription at high phosphorylated NtrC (NtrC~P) concentrations (16). Further complexity at the *glnA* promoter is provided by CRP-cAMP, which can counteract NtrC-dependent regulation by activating *glnAp1* and repressing *glnAp2*, thus integrating C and nitrogen source availability. CRP-cAMP also activates the *glnH* promoter (glutamine transporter) under high-glutamine and low-carbon conditions (14). The mechanisms of CRP-cAMP regulation at those promoters are complex, and we hypothesized that the high α-KG and glutamine levels under glutamine growth could explain the “glutamine paradox” of strong transcriptional activation of *glnA* despite clear glutamine sufficiency. We measured transcript levels of *glnAp1*, *glnA*, and *glnG* by real-time PCR to derive promoter activities of *glnAp1* and *glnAp2* and their relation to *glnG* transcription (Fig. 3B). The overall contribution of *glnAp1* to *glnA* transcription was highest (0.8%) under nitrogen-rich conditions, indicating that nitrogen-dependent transcription control is dominated by NtrC, at least when cells are grown in glucose. The overall *glnA* transcript levels showed an inverse activity in response to the nitrogen status. Notably, *glnA* mRNA levels were lower during glutamine growth than for nitrogen-starved cells, although GS activity measurements suggested the highest output under glutamine (31, 32). The transcription profiles at *glnAp1* and *glnAp2* indicated that both repression and activation, respectively, were dependent on the nitrogen status and NtrC. The lack of transcription driven from *glnAp2* and the elevated transcription levels from *glnAp1* promoters in the Δ*glnG* strain confirmed this. The less well-characterized *glnLp* promoter showed a similar pattern to *glnAp1* when the overall *glnA* transcript levels were taken into account (*glnG*/*glnA*), suggesting that NtrC negatively controls *glnLp* in response to the physiological status. These results indicate that NtrC, in response to nitrogen status, orchestrates expression of both the key nitrogen assimilation protein GS and itself through direct feedback control of *glnAp1*, *glnAp2*, and *glnLp*. While we cannot completely rule out a CRP-mediated mechanism affecting *glnA* expression, it appears that transcription from *glnAp1* under glutamine growth conditions is regulated by NtrC.

### Intracellular concentrations of NtrC and GS.

Phosphorylation of the receiver domain of NtrC shifts the equilibrium from inactive to active NtrC conformers (33). Therefore, transcriptional control by NtrC depends both on NtrC abundance and conformation. NtrB specifically phosphorylates NtrC under nitrogen-limiting conditions but also dephosphorylates NtrC when in complex with nonuridylated PII in a regulated phosphatase reaction. The degree of NtrC phosphorylation depends on the level of glutamine, which determines the uridylylation state of PII, and also on ATP, ADP, and α-KG, which are allosteric effectors modifying PII’s ability to regulate NtrB activities, at least *in vitro* (7).

In order to correlate transcription control with NtrC concentration *in vivo*, we employed a quantitative targeted proteomics approach using multiple-reaction monitoring mass spectroscopy (MRM-MS). MRM-MS allows precise measurements of protein levels and can be used for absolute quantification if combined with a purified isotopically labeled standard (34). This approach provides arguably the most accurate and reliable quantitation of target proteins (35), particularly when using purified labeled proteins as a standard rather than just labeled synthetic peptides (as this also controls for the efficiency of the tryptic digestion step). We established a robust sampling and MRM-MS workflow to determine the intracellular concentrations of NtrC and GS that could be generally applicable for Gram-negative bacteria (see File S1 in the supplemental material for a detailed protocol and quality control). We purified a number of histidine-tagged, ^13^C_6_,^15^N_2_-Lys- and ^13^C_6_,^15^N_4_-Arg-labeled proteins as internal standards (NtrC, GS, IlvE, isocitrate dehydrogenase [IDH], and fumarate and nitrate reductase regulator [FNR]). IlvE, IDH, and FNR served as control proteins to monitor global protein variations that occur in different physiological states but are NtrC independent (8, 36). Intracellular copy numbers have been estimated in *E. coli* under broadly comparable conditions for IDH, FNR, and GS and compare well with our results (37). Protein standard purities for NtrC and GS were 89% and 92% pure, respectively, and isotopic labeling values were 100% and 97%. Across 18 biological samples, total protein extraction in 7 M urea was 92.91% (standard error [SE] of 0.58%), and trypsin digestion efficiency was 89.25% (SE of 1.95%). The main nonsoluble and trypsin-resistant proteins were determined by Edman degradation to be the outer membrane porins OmpF and PhoE (see File S1 in the supplemental material). We conclude that our MRM-MS samples comprised complete peptide sets of NtrC, GS, IlvE, IDH, and FNR derived from the cytoplasmatic *E. coli* proteome. We calculated the copy numbers per cell for each protein based on the reported cell volume of *E. coli* of 1 fl and a cell number of 1.1 × 10^9^/ml/OD_600_ (Table 2) (38) (with 1,000 molecules per cell corresponding to a concentration of 1.61 µM).

**TABLE 2:** Protein concentrations given as copy number/cell under different nitrogen regimes as determined by MRM-MS with protein standard absolute quantification^*a*^

Genotype	N status	Copy no./cell ± SE				
		GS	NtrC	IDH	FNR	IlvE
WT	NH_4_ rich	18,691 ± 536	499 ± 7	25,446 ± 989	8,480 ± 413	8,336 ± 406
WT	Glutamine	66,705 ± 1,084	2,091 ± 78	35,277 ± 1,686	4,183 ± 134	4,116 ± 132
WT	NH_4_ starved	34,681 ± 1,746	1,313 ± 106	29,656 ± 3,540	2,051 ± 49	6,940 ± 544
Δ*glnG*	NH_4_ rich	4,456 ± 221	0 ± 0	31,838 ± 3,345	10,750 ± 947	10,568 ± 931
Δ*glnG*	Glutamine	3,428 ± 183	0 ± 0	46,587 ± 1,426	5,651 ± 49	5,555 ± 230
Δ*glnG*	NH_4_ starved	3,675 ± 171	0 ± 0	48,615 ± 2,677	5,406 ± 694	6,302 ± 190

^a^ GS, glutamine synthetase; NtrC, nitrogen regulation protein C; IDH, isocitrate dehydrogenase; FNR, fumarate and nitrate regulator; IlvE, branched-chain amino acid transferase. SE = 1 standard error of the mean.

GS was most abundant under glutamine growth and least abundant in high ammonium, in line with previous transcriptional reporter gene studies. NtrC was less abundant than GS, supporting a functional role of a putative attenuation site in the *glnA-glnL* intergenic region (32). NtrC was absent in the Δ*glnG* strain, and GS levels were drastically reduced in contrast to IDH, FNR, and IlvE, confirming the dominant and direct role NtrC plays in regulating both *glnA* and itself. For growing cells, *glnA* and *glnG* transcript levels correlated well with GS and NtrC protein levels. NtrC and GS levels in starved cells were lower than those seen for cells grown in glutamine, despite higher *glnA* and *glnG* transcript levels, indicating a higher specific NtrC transcription activity under nitrogen-starved conditions than in glutamine growth.

**FIG 4:**
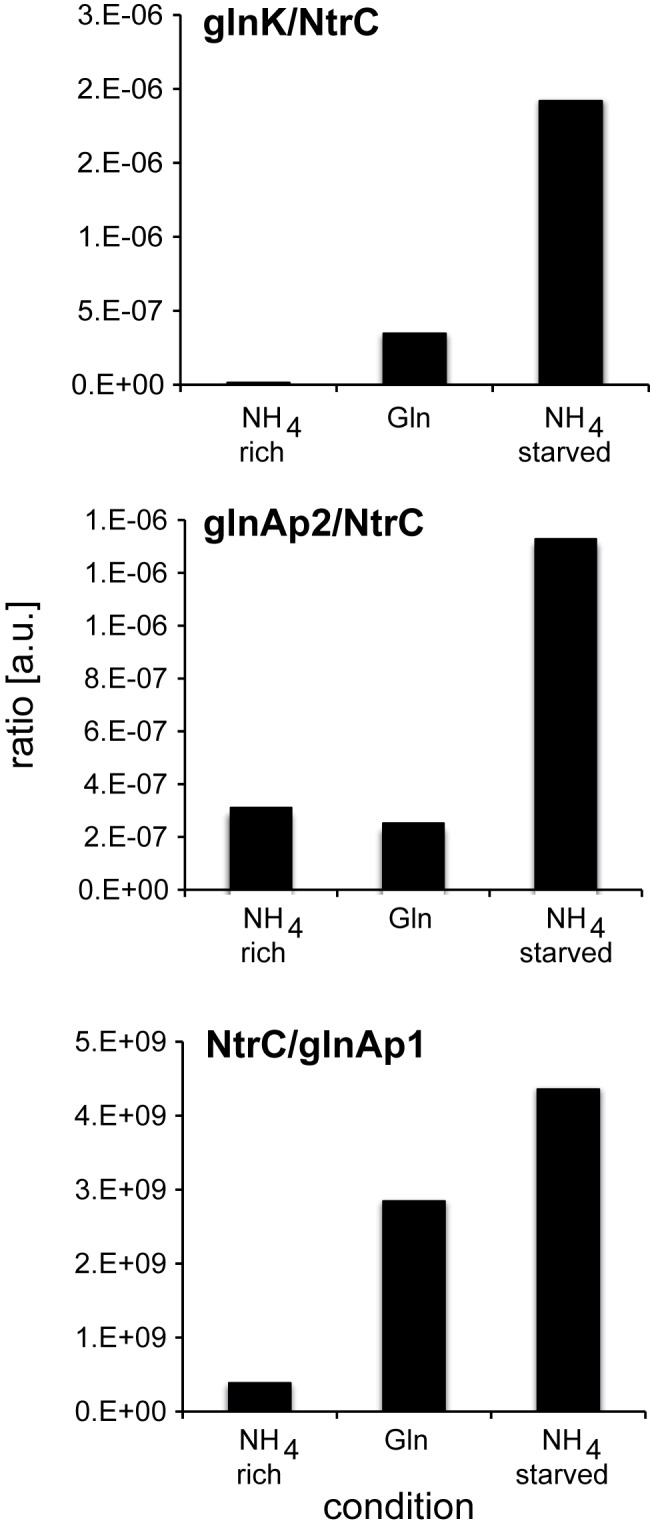
Specific NtrC activation (NtrC^A^) and repression (NtrC^R^) activities at the *glnK*, *glnAp2*, and *glnAp1* promoters. Top and middle, transcript levels from the *glnK* and *plnAp2* promoters per copy number of NtrC molecules reflect on NtrC^A^; bottom, repression activity at *glnAp1* is expressed as NtrC/*glnAp2* expression levels (NtrC^R^). The *y* axis is in arbitrary units.

We could not directly determine the phosphorylation state of NtrC by MRM-MS, probably because NtrC~P is labile (39). However, the transcript/NtrC ratios provide a relative measure of NtrC-specific transcription activity (NtrC^A^), while the inverse relationship (NtrC/transcript) relates to NtrC-specific repression activity (NtrC^R^) at various promoters, as NtrC^A^ depends on adopting an active higher oligomeric state, while NtrC^R^ may not (40, 41). Transcription from the simpler *glnK* promoter depends exclusively on the activation of NtrC through phosphorylation, and therefore transcription levels from *glnKp* in relation to NtrC levels report on NtrC^A^ (42). NtrC^A^ was lowest in nitrogen-rich conditions as expected (Fig. 4A).

Surprisingly, the NtrC^A^ was markedly higher in nitrogen-starved conditions than in glutamine conditions, suggesting that only a subpopulation of NtrC was active in the latter conditions (Fig. 4). This finding is consistent with the high intracellular glutamine levels that would result in PII-mediated repression of NtrC phosphorylation. Notably, NtrC^A^ levels under glutamine growth were higher than NtrC^A^ levels in nitrogen-rich conditions, despite a 5.6-fold-higher glutamine concentration, indicating that signaling controlling formation of NtrC~P depends also on other factors. The glutamine/α-ketoglutarate ratio is thought to determine primarily the phosphorylation state of NtrC (43). However, the glutamine/α-KG ratios in nitrogen-rich, glutamine-starved, and nitrogen-starved conditions were 0.47, 0.76, and 0.057 (Fig. 2), respectively, indicating no simple correlation between these ratios and NtrC^A^.

## DISCUSSION

### NtrC plays a major role in metabolic regulation and adaptation.

The quantitative data on metabolites, proteins, and transcripts allow the assessment of adaptive processes for nitrogen assimilation and regulation in a physiological context. NtrC plays a key role in responding to nitrogen source and nitrogen availability, since growth under all conditions tested here was significantly slower for the Δ*glnG* strain (Table 1) than for the WT strain. When grown with the preferred nitrogen source, ammonium, glutamine levels in the Δ*glnG* strain are at least 35-fold lower than in the WT, which could directly account for slow growth. The low constitutive expression level of *glnA* from the *glnAp1*promoter (Fig. 3) and resulting low level of GS (Table 2) support this interpretation. The cellular nitrogen demand (based on 0.3 pg [dry weight]/cell for *E. coli* and 14% nitrogen content) (44), and assuming that glutamate is exclusively anabolised via the GS/GOGAT cycle when grown in glucose-replete conditions (3), is 1.8 × 10^9^ glutamine molecules per cell. The NCM3722 strain had 18,691 GS molecules under these conditions (Table 2), and assuming a *k*_cat_ value of 50 s^−1^ as determined *in vitro* (45), this would be sufficient to support a generation time of 42 min (producing 2.36 × 10^9^ glutamine molecules/42 min), whereas the fewer GS molecules in the NCM3722Δ*glnG* strain (4,456; producing 5.61 × 10^8^ glutamine molecules/42 min) presumably would not.

One surprising metabolic finding was the high intracellular glutamine level for cells grown on glutamine as a sole nitrogen source (Fig. 2). Therefore, growth under glutamine is not nitrogen limiting in the strict sense of glutamine availability. This observation distinguishes glutamine growth from other nitrogen-limiting nitrogen sources such as aspartate, proline, and arginine, for which intracellular glutamine levels are approximately 10-fold lower than growth on ammonium, at least in the related *Salmonella enterica* serovar Typhimurium (22). The high-affinity glutamine transporter (*glnHPQ*) probably imports most of the glutamine under glutamine growth. The *glnH* promoter architecture (comprising *glnHp1* and *glnHp2*) is very similar to the *glnA* promoter (*glnAp1* and *glnAp2*) (14) and thought to be activated simultaneously by NtrC from the *p1* sites. In agreement with previous findings, we therefore suggest that, similar to GS, the glutamine transporter is highly expressed under glutamine growth (14), thus allowing for rapid glutamine import, since, unlike other amino acid transporters, the high-affinity glutamine transport system (*glnHPQ*) is not repressed by its substrate but by other N-containing compounds, including ammonium (21).

While slow growth and high levels of NtrC-dependent transcription are common on glutamine, aspartate, proline, and arginine, the molecular basis of transcription under glutamine is probably very distinct. For true nitrogen-limiting substrates (e.g., aspartate, arginine), the import and conversion of amino acids to feed into the central nitrogen metabolic pathway, comprising α-KG, glutamate, and glutamine, is likely to be slow, resulting in nitrogen deficiency, low glutamine levels, and phosphorylation of NtrC via UTase, PII, and NtrB signaling. In contrast, under glutamine growth, the conversion of intracellular glutamine into glutamate via GOGAT should be rapid. It is currently unclear if the high α-KG concentrations when grown under glutamine (Fig. 2) are caused by insufficient efflux of α-KG from the Krebs cycle into nitrogen assimilation and/or if GDH deaminates glutamate to α-KG (46). This would in effect constitute a nitrogen assimilation reverse flux from glutamine to α-KG, in which one glutamine and one α-KG would be converted to two glutamate molecules by GOGAT followed by deamination to α-KG by GDH. In either case, the 5.7-fold-higher α-KG level under glutamine growth compared to growth under ammonium-rich conditions (Fig. 2) suggests a direct *in vivo* role for α-KG in regulating signaling to NtrC (see below). Glutamine and α-KG levels were both significantly higher for growth in glutamine than for growth in ammonium, while glutamate levels were the same, suggesting that neither C nor N limitation was responsible for slow growth. As far as we know, neither high intracellular glutamine nor α-KG levels are toxic, and so the slow growth could be a consequence of as-yet-unidentified global effects caused by these or downstream metabolites. For instance, α-KG was recently shown to act as a signal mediating global anabolic and catabolic regulation through inhibition of the production of cyclic AMP and therefore to link α-KG levels with cell growth rates (47). We observed that initial growth of the Δ*glnG* strain in glutamine was particularly slow but reached a growth rate similar to that of the WT after several hours (Fig. 1D), suggesting that slow, presumably global metabolic adaptations to the glutamine regime are involved that are dependent on NtrC.

### Signaling to NtrC dominates transcriptional regulation in response to the nitrogen status.

NtrC^A^ was 400-fold higher at the *glnK* promoter under ammonium-starved than ammonium-rich conditions, providing a numerical *in vivo* approximation of the equilibrium shift induced by NtrB-mediated phosphorylation of NtrC (Fig. 4A). Under ammonium-rich and -starved conditions, we quantified averages of 499 and 1,313 NtrC molecules/cell (0.83 µM and 2.1 µM), respectively. Given the still appreciable number of NtrC molecules present under ammonium-rich conditions, this may suggest that NtrC phosphorylation, rather than increases in copy number, is the determining factor controlling transcription from the *glnK* promoter, at least under our experimental conditions. It has been proposed that as cells run out of ammonium, the strong *glnAp2* promoter would first result in increased NtrC expression, which, above a certain threshold and under inducing conditions, would then activate transcription at the *glnK* and *nac* promoters (16). Recalling that NtrC activity requires the formation of higher oligomers from inactive dimers, and the associated higher-order concentration dependency of such self-association, then NtrC concentration should play an important role in NtrC^A^ formation. However, it is unclear if the 2.5-fold-lower NtrC concentration observed under ammonium-rich than under ammonium-starved conditions would be sufficient to exclude prompt expression of *glnK* following a shift from nitrogen-rich to nitrogen-poor conditions that would not require prior *glnG* expression.

NtrC^A^ at the *glnK* and *glnAp2* promoters was markedly different under ammonium-rich conditions, while NtrC^A^ levels at the *glnAp2* promoter (Fig. 4B) were similar under ammonium-rich and glutamine conditions. Because σ^54^-dependent transcription—in contrast to potential transcription factor-independent transcription from σ^70^-type RNA polymerase (48)—strictly depends on active enhancer binding proteins, we propose that even under these conditions, a subpopulation of NtrC exists in an active conformation. This may imply that the molecular kinetics governing transcription at the *glnAp2* and *glnK* promoters are different, because the total transcript levels at *glnK* and *glnAp2* under ammonium-starved conditions are similar (0.00252 and 0.00174 relative to 16S mRNA, respectively).

The apparent low NtrC^A^ level at the *glnAp2* promoter under glutamine growth could be a direct consequence of nonphosphorylated NtrC repression through binding to the *glnAp2* governor sites p3 and p4, although further experiments would be needed to address this point. NtrC^R^ activities at the *glnAp1* promoter (Fig. 4C), between glutamine and when starved for ammonium, reflect NtrC^A^ activities at the *glnK* and *glnAp2* promoters. This correlation may support an *in vivo* repressor function of NtrC at *glnAp1* that would largely be NtrC~P dependent (32).

### α-KG may override glutamine signaling to NtrC.

We propose that the elevated α-KG level observed under glutamine conditions is, at least in part, directly responsible for the observed high GS and NtrC levels. *In vitro* studies show that α-KG can counteract glutamine signaling by directly binding to PII (7). Although the physiological role of this observation was unclear, the dependency of transcription under glutamine growth conditions with elevated NtrC^A^ activities from various NtrC-dependent promoters indicates that a subpopulation of NtrC must be phosphorylated. We propose that, *in vivo*, elevated α-ketoglutarate concentrations can at least partly override glutamine-dependent signaling to NtrC phosphorylation, most likely through PII binding of α-ketoglutarate resulting in the inhibition of the regulated phosphatase of the PII-NtrB complex on NtrC. As a net result, and contrary to the high intracellular glutamine concentrations when cells are grown with glutamine as the sole nitrogen source, a proportion of NtrC exists in its active, phosphorylated state. Many studies have reported upregulation of NtrC-dependent genes on growth on glutamine as a nitrogen source; the α-ketoglutarate effect would account for this because of the dominant role of NtrC~P in activating the σ^54^ polymerase at various promoters. Our results indicate that NtrC-dependent gene upregulation on glutamine is not because of the hitherto assumed slow glutamine uptake, which would signal nitrogen deficiency, but because high intracellular glutamine results in metabolic fluxes from glutamine to increase the α-KG level, which acts as a metabolic signal of nitrogen regulation.

Our findings could also explain a previously reported inconsistency between the expected results for glutamine, glutamate, and α-KG and NtrC-dependent transcription. Goss and colleagues (49) pointed out that when *E. coli* is deleted for the structural genes for GOGAT (*gltBD*), the glutamine concentrations should be high (since it cannot be depleted rapidly), which could not account for slow growth and the lack of NtrC-dependent activity. When grown on glutamine as a sole nitrogen source, NtrC-dependent activities were highly upregulated (histidase and GS) in WT *E. coli* as expected but not in *gltD* mutants. A high glutamine concentration in a *gltD* mutant could not produce glutamate from glutamine and therefore not reverse nitrogen assimilation flux to α-KG, resulting in derepressed NtrC. Further, growth of the same GOGAT-deficient strains on glutamate as a sole nitrogen source also resulted in highly activated NtrC-dependent activities (49). Neither phenotype (Ntr^−^) could be explained by glutamine starvation inducing the NtrB/NtrC system, but they are fully consistent with high α-KG concentrations derepressing NtrC *in vivo*. Because GOGAT and GDH are NtrC independent, the expectation would be that the α-KG concentrations would also be elevated under glutamine growth in the Δ*glnG* strain, consistent with our measurements (Fig. 2).

## MATERIALS AND METHODS

### Bacterial strains and culturing conditions. 

Strain NCM3722 was used as wild-type *Escherichia coli* (23). The NCM3722Δ*glnG* strain was constructed by transduction using the P1_vir_ bacteriophage as described (50), with JW3839 from the Keio collection serving as the donor strain and NCM3722 as the recipient strain (51). Cells were grown in Gutnick (33.8 mM KH_2_PO_4_, 77.5 mM K_2_HPO_4_, 5.74 mM K_2_SO_4_, 0.41 mM MgSO_4_), supplemented with Ho-LE trace elements (52) and 0.4% glucose, and containing either 10 mM NH_4_Cl (ammonium rich), 3 mM NH_4_Cl (ammonium starved), or 5 mM glutamine. Glutamine was prepared freshly to avoid spontaneous slow hydrolysis into glutamate and ammonia (53). Steady-state samples were taken at OD_600_ values between 0.4 and 0.6. For nitrogen-starved conditions, samples were taken 10 min after growth stopped. We monitored growth until the nitrogen-limiting culture ceased to increase in optical density and sampled the amounts indicated below for the multiomic investigation of nitrogen assimilation regulation. Ammonium concentrations were determined using the Aquaquant ammonium quantification kit (Merck Millipore), according to instructions. All experiments were carried out in three independent biological replicates.

### Transcription assays.

Cell samples were taken from each time point and RNA stabilized with Qiagen RNA protect reagent. Total RNA was extracted with an Invitrogen RNA purification kit and stored in RNase-free water at −80°C. cDNA was generated from 100 ng RNA using the high-capacity cDNA reverse transcription kit (Applied Biosystems). Primer and probe mixtures were custom designed from Invitrogen (TaqMan gene expression Assays). Real-time PCR was performed on an ABI 7500 Fast real-time PCR machine. A 16S RNA gene was chosen as the internal control gene and showed exactly the same expression levels across the conditions tested. The relative expression ratios were calculated using the delta-delta method (PerkinElmer).

### Proteomics.

A detailed proteomics protocol is provided in File S1 in the supplemental material.

### Metabolomics.

Samples for extraction and endometabolome analysis were taken by rapid filtration, using a protocol adopted from Bolten et al. (54) and fully described in Behrends et al. (55). Briefly, 10 ml of cell suspension was harvested by vacuum filtration (filter, PTFE, 0.45-µm pore size, 47 mm diameter, with stand and magnetic filter funnel [Pall, Ann Arbor]) and washed with 5 ml of 1.2× Gutnick medium not containing carbon and nitrogen sources. The filter was then transferred to a precooled (−40°C) 50-ml reaction tube containing 10 ml methanol-acetonitrile-H_2_O (2:2:1, vol/vol/vol) and frozen in liquid nitrogen. The whole procedure took <40 s. To ensure full lysis, all extracts were subjected to two freeze-thaw cycles and sonication. After the removal of the filter, the extracts were centrifuged to pellet the cellular debris, dried in a vacuum concentrator (Eppendorf; 45°C), and resuspended in 1 ml methanol-acetonitrile-H_2_O (2:2:1, vol/vol/vol). Eighty percent of the sample was routinely used for nuclear magnetic resonance (NMR), 10% was used for LC/MS, and 10% was kept as backup. For NMR measurements of the endometabolome, the samples were dried in a vacuum concentrator, resuspended in NMR buffer (90% ^2^H_2_O, 1 mM sodium 2,2-dimethyl-2-silapentane-^2^H_6_-5-sulfonate [DSS], 5 mM NaN_3_), and transferred to a 5-mm NMR tube. For LC/MS analysis, samples were dried in a vacuum concentrator, resuspended in 50 µl methanol-acetonitrile-H_2_O (2:2:1, vol/vol/vol), and mixed with 25 µl of 15 µM ^13^C_5_,^15^N_2_-glutamine in the same solvent for quantification. For analysis of the exometabolome, samples were obtained by centrifuging 500 µl of culture (16,000 × *g*, 1 min, room temperature). A total of 480 µl of the supernatant was mixed with 120 µl buffer (5 mM DSS and 25 mM NaN_3_ in 100% ^2^H_2_O).

### Nuclear magnetic resonance spectroscopy. 

Spectra were acquired on a Bruker Avance DRX600 NMR spectrometer (Bruker BioSpin, Rheinstetten, Germany), with a magnetic field strength of 14.1 T and resulting ^1^H resonance frequency of 600 MHz, equipped with a 5-mm inverse probe following an approach described in Beckonert et al. (56). One-dimensional spectra of extracts were routinely acquired with 768 transients with 8 dummy scans using a standard NOESYPR1d water suppression pulse sequence, while supernatants were acquired with 64 scans. After acquisition, spectra were Fourier transformed and phased in iNMR (Nucleomatica, Molfetta, Italy). The full-resolution data were exported as ASCII and imported into Matlab (MathWorks) using an in-house code for further analysis. Peaks were assigned using spectral information from previous studies (57) and the BioMagResBank online database (58).

### Liquid chromatography mass spectrometry.

LC/MS was performed using a method adapted from Spagou et al. (59). Briefly, the samples were chromatographed on an Acquity UPLC (ultra-performance liquid chromatography) system with an Acquity UPLC BEH HILIC (1.7-µm, 2.1- by 100-mm) column (Waters Corp., Milford, MA) at 40°C. Separation of glutamine from glutamate and α-ketoglutarate was achieved with gradient elution of 90/10% ACN-H_2_O (A) and 50/50% ACN-H_2_O (B), both containing 0.1% (vol/vol) formic acid and 10 mM ammonium acetate, at a flow rate of 0.4 ml/min. Starting conditions were 99.0% A and 1% B for 1.0 min, changing linearly to 100% B over the next 11 min, after which the solvent composition returned to starting conditions over 0.1 min, followed by reequilibration for 3.9 min prior to the next injection. Spectra were acquired in negative mode on a Waters Xevo TQ-S tandem quadrupole mass spectrometer. Monitored parent-to-fragment ion transitions were 147 to >84 for glutamine, 148 to >84 for glutamate, and 154 to >89 for ^13^C_5_,^15^N_2_-glutamine. The cone voltage and collision energy were set to 25 V and 15 V, respectively. Data were exported as CDF files and integrated in Matlab using in-house code based on Behrends et al. (60).

## SUPPLEMENTAL MATERIAL

Figure S1 Growth and ammonium consumption of NCM3733 in ammonium-rich and ammonium-poor media. Following inoculation into defined media with initial NH_4_Cl concentrations of 10 mM and 3 mM, growth over time (*x* axis) was measured by OD_600_ and NH_4_Cl concentrations measured from the supernatants to determine consumption (secondary axis), using Aquaquant. Red and black circles show OD_600_ and NH_4_Cl values for growth on 3 mM NH_4_Cl, respectively. Blue and green circles indicate OD_600_ and NH_4_Cl for growth in 10 mM NH_4_Cl. Download Figure S1, TIF file, 0.1 MB

Figure S2 NMR spectra of culture supernatant and cell extract grown in 5 mM Gln and 0.4% glucose. Compared to the supernatant fraction, the lack of detectable glucose in the cell extract fraction indicates no or negligible carryover of medium components into the cell extract fraction. Intensity levels were normalized to the levels of the Gln multiplet at δ2.45 ppm. Ace, acetate; Gln, glutamine; Glu, glutamate; Glc, glucose; Glcα, glucose, α-anomeric proton; Glcβ, glucose, β-anomeric proton; Tre, trehalose. Download Figure S2, TIF file, 0.1 MB

File S1 Additional protocols. Download File S1, PDF file, 0.8 MB

## References

[1] Reitzer L 2003 Nitrogen assimilation and global regulation in *Escherichia coli*. Annu. Rev. Microbiol. 57:155–176 12730324 10.1146/annurev.micro.57.030502.090820

[2] Yan D 2007 Protection of the glutamate pool concentration in enteric bacteria. Proc. Natl. Acad. Sci. U. S. A. 104:9475–9480 17517610 10.1073/pnas.0703360104 PMC1890519

[3] Helling RB 1994 Why does *Escherichia coli* have two primary pathways for synthesis of glutamate? J. Bacteriol. 176:4664–4668 7913929 10.1128/jb.176.15.4664-4668.1994 PMC196288

[4] Brown MS Segal A Stadtman ER 1971 Modulation of glutamine synthetase adenylylation and deadenylylation is mediated by metabolic transformation of the P II -regulatory protein. Proc. Natl. Acad. Sci. U. S. A. 68:2949–2953 4399832 10.1073/pnas.68.12.2949 PMC389567

[5] Okano H Hwa T Lenz P Yan D 2010 Reversible adenylylation of glutamine synthetase is dynamically counterbalanced during steady-state growth of *Escherichia coli*. J. Mol. Biol. 404:522–536 20887734 10.1016/j.jmb.2010.09.046

[6] Jiang P Ninfa AJ 1999 Regulation of autophosphorylation of *Escherichia coli* nitrogen regulator II by the PII signal transduction protein. J. Bacteriol. 181:1906–1911 10074086 10.1128/jb.181.6.1906-1911.1999 PMC93592

[7] Jiang P Ninfa AJ 2009 Alpha-ketoglutarate controls the ability of the *Escherichia coli* PII signal transduction protein to regulate the activities of NRII (NrB) but does not control the binding of PII to NRII. Biochemistry 48:11514–11521 19877669 10.1021/bi901158h PMC2786246

[8] Zimmer DP Soupene E Lee HL Wendisch VF Khodursky AB Peter BJ Bender RA Kustu S 2000 Nitrogen regulatory protein C-controlled genes of *Escherichia coli*: scavenging as a defense against nitrogen limitation. Proc. Natl. Acad. Sci. U. S. A. 97:14674–14679 11121068 10.1073/pnas.97.26.14674 PMC18977

[9] Rombel I North A Hwang I Wyman C Kustu S 1998 The bacterial enhancer-binding protein NtrC as a molecular machine. Cold Spring Harb. Symp. Quant. Biol. 63:157–166 10384279 10.1101/sqb.1998.63.157

[10] Schumacher J Zhang X Jones S Bordes P Buck M 2004 ATP-dependent transcriptional activation by bacterial PspF AAA+ protein. J. Mol. Biol. 338:863–875 15111053 10.1016/j.jmb.2004.02.071

[11] Rappas M Schumacher J Niwa H Buck M Zhang X 2006 Structural basis of the nucleotide driven conformational changes in the AAA+ domain of transcription activator PspF. J. Mol. Biol. 357:481–492 16430918 10.1016/j.jmb.2005.12.052

[12] Wigneshweraraj S Bose D Burrows PC Joly N Schumacher J Rappas M Pape T Zhang X Stockley P Severinov K Buck M 2008 Modus operandi of the bacterial RNA polymerase containing the sigma54 promoter-specificity factor. Mol. Microbiol. 68:538–546 18331472 10.1111/j.1365-2958.2008.06181.x

[13] Ninfa AJ Magasanik B 1986 Covalent modification of the *glnG* product, NRI, by the *glnL* product, NRII, regulates the transcription of the *glnALG* operon in *Escherichia coli*. Proc. Natl. Acad. Sci. U. S. A. 83:5909–5913 2874557 10.1073/pnas.83.16.5909 PMC386406

[14] Mao XJ Huo YX Buck M Kolb A Wang YP 2007 Interplay between CRP-cAMP and PII-Ntr systems forms novel regulatory network between carbon metabolism and nitrogen assimilation in *Escherichia coli*. Nucleic Acids Res. 35:1432–1440 17284458 10.1093/nar/gkl1142 PMC1865078

[15] Atkinson MR Ninfa AJ 1998 Role of the GlnK signal transduction protein in the regulation of nitrogen assimilation in *Escherichia coli*. Mol. Microbiol. 29:431–447 9720863 10.1046/j.1365-2958.1998.00932.x

[16] Atkinson MR Pattaramanon N Ninfa AJ 2002 Governor of the glnAp2 promoter of *Escherichia coli*. Mol. Microbiol. 46:1247–1257 12453212 10.1046/j.1365-2958.2002.03211.x

[17] van Heeswijk WC Molenaar D Hoving S Westerhoff HV 2009 The pivotal regulator GlnB of *Escherichia coli* is engaged in subtle and context-dependent control. FEBS J. 276:3324–3340 19438718 10.1111/j.1742-4658.2009.07058.x

[18] Shiau SP Schneider BL Gu W Reitzer LJ 1992 Role of nitrogen regulator I (NtrC), the transcriptional activator of *glnA* in enteric bacteria, in reducing expression of *glnA* during nitrogen-limited growth. J. Bacteriol. 174:179–185 1345910 10.1128/jb.174.1.179-185.1992 PMC205693

[19] Sasse-Dwight S Gralla JD 1988 Probing the *Escherichia coli glnALG* upstream activation mechanism in vivo. Proc. Natl. Acad. Sci. U. S. A. 85:8934–8938 2904147 10.1073/pnas.85.23.8934 PMC282621

[20] Bender RA Magasanik B 1977 Regulatory mutations in the Klebsiella aerogenes structural gene for glutamine synthetase. J. Bacteriol. 132:100–105 21157 10.1128/jb.132.1.100-105.1977 PMC221831

[21] Willis RC Iwata KK Furlong CE 1975 Regulation of glutamine transport in *Escherichia coli*. J. Bacteriol. 122:1032–1037 238938 10.1128/jb.122.3.1032-1037.1975 PMC246156

[22] Ikeda TP Shauger AE Kustu S 1996 Salmonella typhimurium apparently perceives external nitrogen limitation as internal glutamine limitation. J. Mol. Biol. 259:589–607 8683567 10.1006/jmbi.1996.0342

[23] Soupene E van Heeswijk WC Plumbridge J Stewart V Bertenthal D Lee H Prasad G Paliy O Charernnoppakul P Kustu S 2003 Physiological studies of *Escherichia coli* strain MG1655: growth defects and apparent cross-regulation of gene expression. J. Bacteriol. 185:5611–5626 12949114 10.1128/JB.185.18.5611-5626.2003 PMC193769

[24] Bennett BD Kimball EH Gao M Osterhout R Van Dien SJ Rabinowitz JD 2009 Absolute metabolite concentrations and implied enzyme active site occupancy in *Escherichia coli*. Nat. Chem. Biol. 5:593–599 19561621 10.1038/nchembio.186 PMC2754216

[25] Rothstein DM Pahel G Tyler B Magasanik B 1980 Regulation of expression from the *glnA* promoter of *Escherichia coli* in the absence of glutamine synthetase. Proc. Natl. Acad. Sci. U. S. A. 77:7372–7376 6111793 10.1073/pnas.77.12.7372 PMC350505

[26] Thakur CS Brown ME Sama JN Jackson ME Dayie TK 2010 Growth of wildtype and mutant *E. coli* strains in minimal media for optimal production of nucleic acids for preparing labeled nucleotides. Appl. Microbiol. Biotechnol. 88:771–779 20730533 10.1007/s00253-010-2813-y PMC2938442

[27] Kashket ER Brodie AF 1962 Effects of near-ultraviolet irradiation on growth and oxidative metabolism of bacteria. J. Bacteriol. 83:1094–1100 14454145 10.1128/jb.83.5.1094-1100.1962 PMC279412

[28] Vijayendran C Polen T Wendisch VF Friehs K Niehaus K Flaschel E 2007 The plasticity of global proteome and genome expression analyzed in closely related W3110 and MG1655 strains of a well-studied model organism, *Escherichia coli*-K12. J. Biotechnol. 128:747–761 17331609 10.1016/j.jbiotec.2006.12.026

[29] Maharjan RP Ferenci T 2005 Metabolomic diversity in the species *Escherichia coli* and its relationship to genetic population structure. Metabolomics 1:235–242

[30] Kim M Zhang Z Okano H Yan D Groisman A Hwa T 2012 Need-based activation of ammonium uptake in *Escherichia coli*. Mol. Syst. Biol. 8:616 23010999 10.1038/msb.2012.46 PMC3472687

[31] Reitzer LJ Magasanik B 1986 Transcription of *glnA* in *E. coli* is stimulated by activator bound to sites far from the promoter. Cell 45:785–792 2871943 10.1016/0092-8674(86)90553-2

[32] Pahel G Rothstein DM Magasanik B 1982 Complex *glnA-glnL-glnG* operon of *Escherichia coli*. J. Bacteriol. 150:202–213 6120929 10.1128/jb.150.1.202-213.1982 PMC220100

[33] Volkman BF Lipson D Wemmer DE Kern D 2001 Two-state allosteric behavior in a single-domain signaling protein. Science 291:2429–2433 11264542 10.1126/science.291.5512.2429

[34] Picotti P Aebersold R 2012 Selected reaction monitoring-based proteomics: workflows, potential, pitfalls and future directions. Nat. Methods 9:555–566 22669653 10.1038/nmeth.2015

[35] Elschenbroich S Kislinger T 2011 Targeted proteomics by selected reaction monitoring mass spectrometry: applications to systems biology and biomarker discovery. Mol. Biosyst. 7:292–303 20976349 10.1039/c0mb00159g

[36] Ishihama Y Schmidt T Rappsilber J Mann M Hartl FU Kerner MJ Frishman D 2008 Protein abundance profiling of the *Escherichia coli* cytosol. BMC Genomics 9:102 18304323 10.1186/1471-2164-9-102 PMC2292177

[37] Lu P Vogel C Wang R Yao X Marcotte EM 2007 Absolute protein expression profiling estimates the relative contributions of transcriptional and translational regulation. Nat. Biotechnol. 25:117–124 17187058 10.1038/nbt1270

[38] Kubitschek HE Friske JA 1986 Determination of bacterial cell volume with the Coulter counter. J. Bacteriol. 168:1466–1467 3536882 10.1128/jb.168.3.1466-1467.1986 PMC213663

[39] Kamberov ES Atkinson MR Chandran P Ninfa AJ 1994 Effect of mutations in *Escherichia coli glnL* (*ntrB*), encoding nitrogen regulator II (NRII or NtrB), on the phosphatase activity involved in bacterial nitrogen regulation. J. Biol. Chem. 269:28294–28299 7961767

[40] Yan D Kustu S 1999 “Switch I” mutant forms of the bacterial enhancer-binding protein NtrC that perturb the response to DNA. Proc. Natl. Acad. Sci. U. S. A. 96:13142–13146 10557287 10.1073/pnas.96.23.13142 PMC23914

[41] De Carlo S Chen B Hoover TR Kondrashkina E Nogales E Nixon BT 2006 The structural basis for regulated assembly and function of the transcriptional activator NtrC. Genes Dev. 20:1485–1495 16751184 10.1101/gad.1418306 PMC1475761

[42] Atkinson MR Blauwkamp TA Bondarenko V Studitsky V Ninfa AJ 2002 Activation of the *glnA*, *glnK*, and *nac* promoters as *Escherichia coli* undergoes the transition from nitrogen excess growth to nitrogen starvation. J. Bacteriol. 184:5358–5363 12218022 10.1128/JB.184.19.5358-5363.2002 PMC135341

[43] Magasanik B 1989 Regulation of transcription of the *glnALG* operon of *Escherichia coli* by protein phosphorylation. Biochimie 71:1005–1012 2574599 10.1016/0300-9084(89)90104-1

[44] Neidhardt FC Curtiss R III Ingraham JL Lin EC Low KB Magasanik B Reznikoff WS Riley M Schaechter M Umbarger HE 1996 *Escherichia coli* and *Salmonella*: cellular and molecular biology, 2nd ed. ASM Press, Washington, DC

[45] Witmer MR Palmieri-Young D Villafranca JJ 1994 Probing the catalytic roles of n2-site glutamate residues in *Escherichia coli* glutamine synthetase by mutagenesis. Protein Sci. 3:1746–1759 7849593 10.1002/pro.5560031015 PMC2142605

[46] Sharkey MA Oliveira TF Engel PC Khan AR 2013 Structure of NADP-dependent glutamate dehydrogenase from *Escherichia coli*: reflections on the basis of coenzyme specificity in the family of glutamate dehydrogenases. FEBS J. 280 (18)**:** 4681–4692. 10.1111/febs.12439 23879525 PMC3809065

[47] You C Okano H Hui S Zhang Z Kim M Gunderson CW Wang YP Lenz P Yan D Hwa T 2013 Coordination of bacterial proteome with metabolism by cyclic AMP signalling. Nature 500:301–306 23925119 10.1038/nature12446 PMC4038431

[48] Buck M Bose D Burrows P Cannon W Joly N Pape T Rappas M Schumacher J Wigneshweraraj S Zhang X 2006 A second paradigm for gene activation in bacteria. Biochem. Soc. Trans. 34:1067–1071 17073752 10.1042/BST0341067

[49] Goss TJ Perez-Matos A Bender RA 2001 Roles of glutamate synthase, *gltBD*, and *gltF* in nitrogen metabolism of *Escherichia coli* and *Klebsiella aerogenes*. J. Bacteriol. 183:6607–6619 11673431 10.1128/JB.183.22.6607-6619.2001 PMC95492

[50] Miller JH 1992 A short course in bacterial genetics: a laboratory manual and handbook for *Escherichia coli* and related bacteria. Cold Spring Harbor Laboratory Press, Cold Spring Harbor, NY

[51] Baba T Ara T Hasegawa M Takai Y Okumura Y Baba M Datsenko KA Tomita M Wanner BL Mori H 2006 Construction of *Escherichia coli* K-12 in-frame, single-gene knockout mutants: the Keio collection. Mol. Syst. Biol. 2:2006 0008 10.1038/msb4100050 PMC1681482 16738554

[52] Atlas RM 2010 Handbook of microbiological media, vol 1 CRC Press, Boca Raton, FL

[53] Bender RA Macaluso A Magasanik B 1976 Glutamate dehydrogenase: genetic mapping and isolation of regulatory mutants of *Klebsiella aerogenes*. J. Bacteriol. 127:141–148 6429 10.1128/jb.127.1.141-148.1976 PMC233044

[54] Bolten CJ Kiefer P Letisse F Portais JC Wittmann C 2007 Sampling for metabolome analysis of microorganisms. Anal. Chem. 79:3843–3849 17411014 10.1021/ac0623888

[55] Behrends V Williams KJ Jenkins VA Robertson BD Bundy JG 2012 Free glucosylglycerate is a novel marker of nitrogen stress in *Mycobacterium smegmatis*. J. Proteome Res. 11:3888–3896 22650367 10.1021/pr300371b

[56] Beckonert O Keun HC Ebbels TM Bundy J Holmes E Lindon JC Nicholson JK 2007 Metabolic profiling, metabolomic and metabonomic procedures for NMR spectroscopy of urine, plasma, serum and tissue extracts. Nat. Protoc. 2:2692–2703 18007604 10.1038/nprot.2007.376

[57] Behrends V Ebbels TM Williams HD Bundy JG 2009 Time-resolved metabolic footprinting for nonlinear modeling of bacterial substrate utilization. Appl. Environ. Microbiol. 75:2453–2463 19218401 10.1128/AEM.01742-08 PMC2675220

[58] Ulrich EL Akutsu H Doreleijers JF Harano Y Ioannidis YE Lin J Livny M Mading S Maziuk D Miller Z Nakatani E Schulte CF Tolmie DE Kent Wenger R Yao H Markley JL 2008 BioMagResBank. Nucleic Acids Res. 36:D402–D408 17984079 10.1093/nar/gkm957 PMC2238925

[59] Spagou K Wilson ID Masson P Theodoridis G Raikos N Coen M Holmes E Lindon JC Plumb RS Nicholson JK Want EJ 2011 HILIC-UPLC-MS for exploratory urinary metabolic profiling in toxicological studies. Anal. Chem. 83:382–390 21142126 10.1021/ac102523q

[60] Behrends V Tredwell GD Bundy JG 2011 A software complement to AMDIS for processing GC-MS metabolomic data. Anal. Biochem. 415:206–208 21575589 10.1016/j.ab.2011.04.009

