# Supplementary figures and images for: Nitrogen and Carbon Status Are Integrated at the Transcriptional Level by the Nitrogen Regulator NtrC In Vivo
Source: mBio. 2013 Nov 19;4(6):e00881-13. doi: 10.1128/mBio.00881-13 (PMC3870243; doi:10.1128/mBio.00881-13)

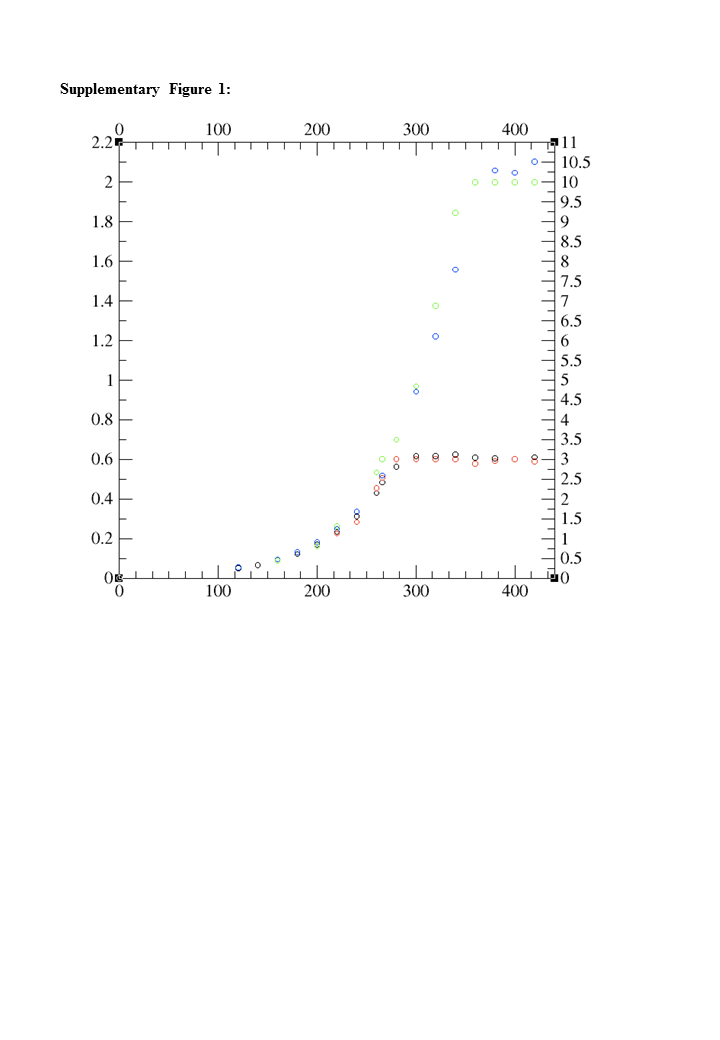

Supplement: Figure S1 — Growth and ammonium consumption of NCM3733 in ammonium-rich and ammonium-poor media. Following inoculation into defined media with initial NH4Cl concentrations of 10 mM and 3 mM, growth over time (x axis) was measured by OD600 and NH4Cl concentrations measured from the supernatants to determine consumption (secondary axis), using Aquaquant. Red and black circles show OD600 and NH4Cl values for growth on 3 mM NH4Cl, respectively. Blue and green circles indicate OD600 and NH4Cl for growth in 10 mM NH4Cl. Download [file mbo006131675sf01.tif]

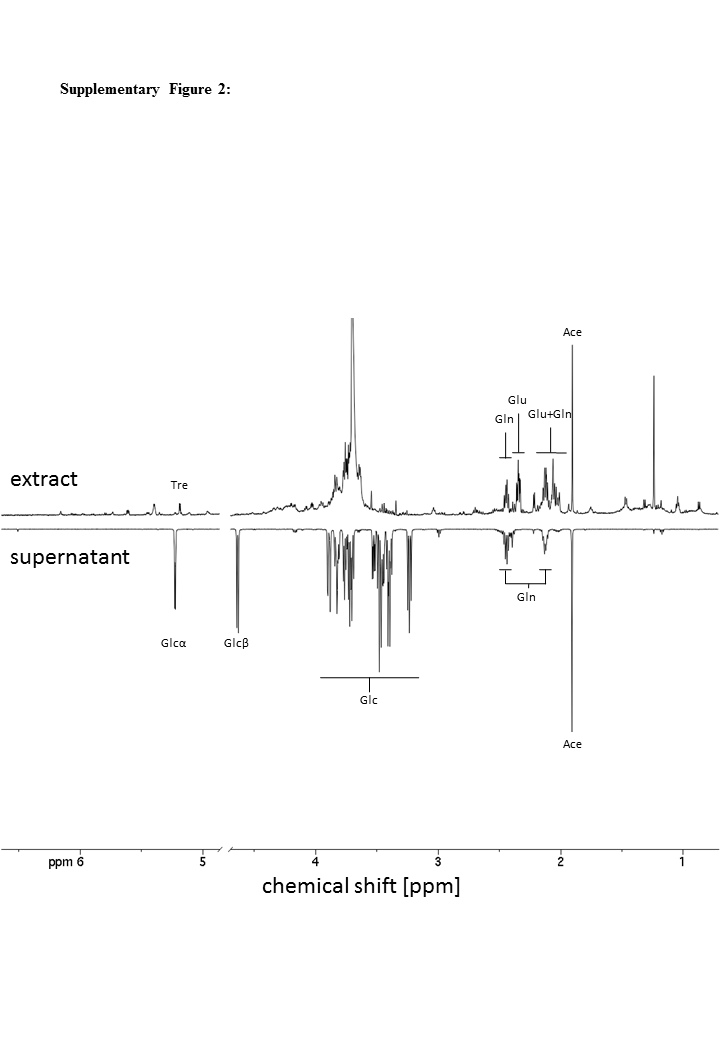

Supplement: Figure S2 — NMR spectra of culture supernatant and cell extract grown in 5 mM Gln and 0.4% glucose. Compared to the supernatant fraction, the lack of detectable glucose in the cell extract fraction indicates no or negligible carryover of medium components into the cell extract fraction. Intensity levels were normalized to the levels of the Gln multiplet at δ2.45 ppm. Ace, acetate; Gln, glutamine; Glu, glutamate; Glc, glucose; Glcα, glucose, α-anomeric proton; Glcβ, glucose, β-anomeric proton; Tre, trehalose. Download [file mbo006131675sf02.tif]
