# Supplementary material for: Nitrogen and Carbon Status Are Integrated at the Transcriptional Level by the Nitrogen Regulator NtrC In Vivo
Source: mBio. 2013 Nov 19;4(6):e00881-13. doi: 10.1128/mBio.00881-13 (PMC3870243; doi:10.1128/mBio.00881-13)
Supplement: File S1 — Additional protocols. Download [file mbo006131675s1.pdf]

Multiple Reaction Monitoring Mass Spectroscopy with PSAQ

The reproducible quantification of proteins from complex biological samples depends on the robustness of sample preparation and measurements. We have developed and evaluated a workflow for the extraction of *Escherichia coli* high urea soluble proteins and quantification of targeted proteins using multiple reaction monitoring mass spectroscopy (MRM-MS) in conjunction with protein standard absolute quantification (PSAQ).

Advances in multiple reaction monitoring mass spectroscopy MRM-MS, can be applied to quantify targeted proteins in complex biological samples with high sensitivity (reviewed [1]). This approach does not require pre-separation of proteins from biological samples as is commonly done using 2D gel electophoresis [2]. Instead the pre-digested peptides are eluted from a liquid chromatography in-line with the ionisation source of the mass spectrometer (LC-MS). Using the unique capacity of triple quadrupole mass spectrometers, where the three chambers (Q1, Q2, Q3) can be set to act as mass filter, collision chamber and fragment ion detector, selected precursor-fragment ion pairs (termed transitions) can be measured directly. The addition of isotopically labelled purified full length protein standards of known concentration to the sample of interest allows the absolute quantification of targeted sample proteins in MRM-MS (PSAQ). PSAQ was proposed to offer a number of advantages compared to other isotopic internal standard strategies (reviewed [3]). With the addition of the PSAQ standard to the sample (Figure 1), the biochemical identity of the labelled full length protein with that of the analyte protein internally corrects for any losses during sample handling and incomplete trypsin digestion.

For the determination of intracellular protein concentrations we optimised sample preparation and measurements in order to increase reproducibility across biological samples. For the results shown in table two of the main document, standard errors of the mean across three biological replicates ranged from 0.8% to 13%, demonstrating high robustness of the workflow.

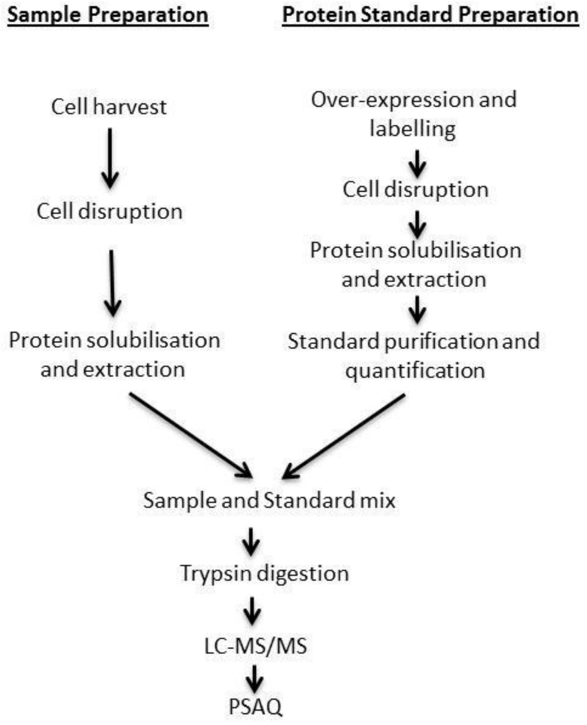

Figure 1: MRM-MS and PSAQ workflow.

Sample Preparation:

Bacterial batch cultures were grown under aerobic conditions in Gutnick medium (33.8 mM KH_2PO_4, 77.5 mM K_2HPO_4, 5.74 mM K_2SO_4, 0.41 mM $MgSO_4$), supplemented with Ho-LE trace elements (Handbook of Microbiological Media, Volume 1, Ronald, M., Atlas), 0.4% glucose and different initial N source conditions: 10 mM NH_4Cl, 5 mM L-glutamine, 3 mM NH_4Cl. 10 ml samples were extracted during logarithmic growth between OD600 of 0.4-0.6 or 10 minutes after growth arrest when grown in 3 mM NH_4Cl. Cells were harvested by 20 minutes centrifugation at 5000xg at 4˚C. For cell disruption and protein extraction, pellets were resuspended in 1ml 1x Gutnick medium, 7M urea, 1mM TCEP. Re-suspended pellets were disrupted on ice by sonication for a discontinuous 7.5 minutes using a VWR7000 sonicator with 40% pulse. Extracted urea soluble proteins were recovered in the supernatant fraction following centrifugation at 15000 x g for 45 minutes at 4˚C. For control purposes we re-suspended the pellet in 1ml 1x Gutnick medium with 7M Urea by rigorous shaking and vortexing. To evaluate protein extraction efficiencies for all samples, 10□l of the re-suspended bacterial pellet (Figure 2, c1), the extracted protein fraction (c2) and the re-suspended cell debris fraction (c3) were analysed by SDS-PAGE. Proteins were stained by SYPRO® Ruby Protein Gel Stain (Invitrogen). SDS-PAGE gels were imaged using Fuji FLA-5000 PhosphorImager with a Fluor stage 4046, FITC filter and wavelength 473 nm. Proteins were quantified by fluorescence using the Aida software. Total protein extraction efficiencies were 92.91% (SE 0.58%), indicating good yield and reproducibility. We found that 7M urea greatly increased disruption and solubilisation efficiencies and prolonged sonication improved reproducibility.

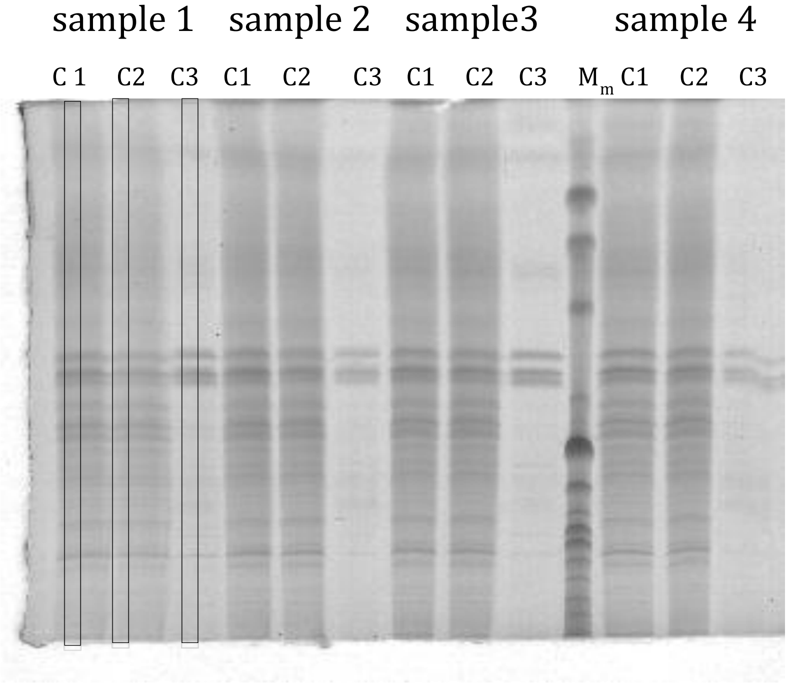

Figure 2: Examples of SYPRO® Ruby stained and visualised SDS-PAGE for estimating protein extraction efficiencies. Total fluorescence intensities of rectangular regions were determined (as shown in lanes 1-3) for each lane and extraction efficiencies per sample calculated from band intensities.

Correlation between OD^{600} and extracted protein amounts

Because cellular nitrogen is an important component of proteins, protein availability may impact on global protein production. Further, different nitrogen availability may also impact on cell size and therefore on OD^{600} cell density estimates. Optical density measurements provide a convenient method to determine bacterial cell densities. For *Escherichia coli* grown in minimal glucose media cell densities are commonly approximated as $1.1* 10^9$cells/ml for OD^{600} = 1. However, cell volumes are growth rate dependent and can impact on OD^{600} based estimates of cell numbers [4]. We therefore correlated OD^{600} and total extracted protein concentrations (Figure 3). While these independent measurements do not exclude the possibility of different cell sizes, the good correlation shown in Figure 3 indicates that the determined intracellular protein concentrations represent good relative estimates with respect to total protein abundance in our samples.

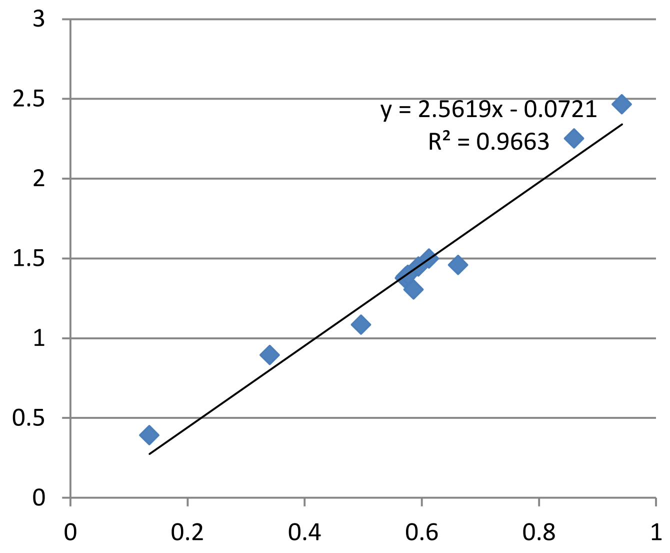

Figure 3: Correlation between OD^{600} and of protein extract concentrations during logarithmic growth, determined by Bradford protein estimation.

Protein standard preparation:

Protein standards for PSAQ were doubly labelled at arginine (L-Arginine- $^{13}C_6$, $^{15}N_4$, Sigma-Aldrich) and lysine (L-Lysine-$^{13}C_6$, $^{15}N_2$, Sigma-Aldrich) residues *in vivo.* NCM3722 were transformed with AG1(pCA24N, -gfp) plasmids from the Aska(-) collection (DNA Research 12, 291-299(2005)) and plated on LB chloramphenicol (50 µg/ml). The Aska (-) collection provides clones harbouring expression plasmids for nearly all *E.coli* structural genes and we obtained AG1(pCA24N, -gfp) derivatives to over-express histidine tagged, NtrC (JW3839), GS (JW3841), IlvE (JW5606), IDH (JW1122), FNR(JW1328). Overnight cultures were grown in Gutnick minimal media with 0.4% glucose and 10mM NH_4Cl, at 37˚C, comprising labelled arginine and lysine and eighteen unlabelled amino acids at 1mM concentrations. Overnight cultures were diluted 50 fold for 100ml day cultures grown in same media. When cultures reached an OD^{600} of 0.5, Isopropyl-β-D- thiogalactopyranosid (IPTG) was added to a final 1 mM and cultures grown for further 4 h. Cells were harvested by centrifugation at 4°C, 4000 rpm, for 20 min and pellets were stored at -20°C. Cell pellets were re-suspended in 10ml 1x Gutnick medium with 7M urea and cells disrupted by sonication for a discontinuous 7.5 minutes using a VWR7000 sonicator with 40% pulse, followed by centrifugation at 15000 x g, 45 minutes at 4˚C.

Histidine tagged proteins were purified from the soluble fraction by nickel affinity chromatography on an Äkta FPLC system (General Electrics). The soluble fractions were loaded onto 1ml HisTrap HP columns (General Electrics) and the column washed extensively with binding buffer (20mM sodium phosphate, 0.5M NaCl, pH 7.4, 7M urea) and column bound proteins were eluted during a 20ml imidazole gradient (from 0 to 200 mM) at 1ml/min flow rate in an otherwise identical buffer as the binding buffer. Fractions containing the desired proteins, as judged by SDS-PAGE, were pooled and dialysed against 1x Gutnick, 7M urea, 50% glycerol, 1mM TCEP (Tris(2-carboxyethyl)phosphine), for storage at -80C and to prevent oxidation.

Protein standard quantification and quality control

Protein standard concentrations were estimated using the Bradford assay based Bio-Rad protein assay (Bio-Rad), following the manufacturer's instructions and using BSA as protein standard. Protein standard purities were estimated by relative band fluorescence intensities of SYPRO® Ruby stained SDS-PAGE gels, using the Aida software (Figure 4).

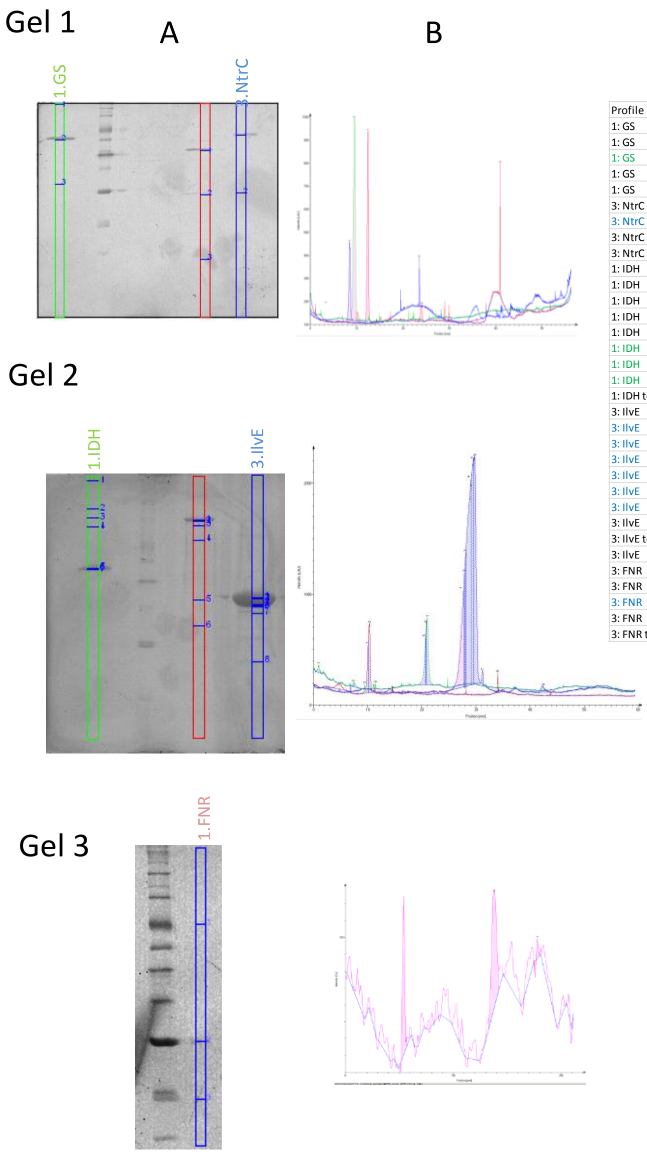

Profile	Peak	Integral [	Bkg [LAU]	Integral-Bkg [LA	**% purity**
1: GS					
1: GS	1	34313	34127	186.5416	
1: GS	2	34083	16235	17847.501	
1: GS	3	31684	31391	293.2066	
1: GS				18327.2492	**97.38**
3: NtrC					
3: NtrC	1	16637	11330	5306.6146	
3: NtrC	2	6571	5930	640.9156	
3: NtrC total				5947.5302	**89.22**
1: IDH					
1: IDH	1	14244	13283	961.6204	
1: IDH	2	2544	2385	159.2719	
1: IDH	3	1365	1331	34.3386	
1: IDH	4	1711	1645	66.5083	
1: IDH	5	13879	8909	4969.4458	
1: IDH	6	4868	1269	3599.4542	
1: IDH	7	17240	7413	9826.7104	18395.61
1: IDH total				19617.3496	**93.77**
3: IlvE					
3: IlvE	1	77013	54795	22217.9202	
3: IlvE	2	7951	1158	6793.5212	
3: IlvE	3	11735	1511	10223.8852	
3: IlvE	4	71515	7207	64307.6317	
3: IlvE	5	31989	2769	29220.2309	
3: IlvE	6	56581	10145	46436.6967	179199.9
3: IlvE	7	7031	5679	1351.2217	
3: IlvE total	8	56116	53998	2118.8411	
3: IlvE				182669.9487	**98.10**
3: FNR					
3: FNR	1	3964	3777	186.8909	
3: FNR	2	6272	5904	367.6211	
3: FNR	3	3433	3435	-2.1	
3: FNR total				552.412	**66.55**

Figure 4: Estimation of protein standard purities: A) SYPRO® Ruby stained SDS-PAGE gel fluorescence images and showing traces for 1D evaluation. B) Lane band intensity profiles for 1D evaluation for analysed lanes, colour coded as in A (red traces in gels 1 and 2 are not relevant for this work). C) Band peak intensities of profiles in B where used to calculate protein purities by dividing the sum of peak intensities corresponding to the molecular masses of the proteins of interest, divided by the sum of total peak intensities of the lane. In the case of the low purity FNR protein, calculations of protein abundance following MRM-MS were corrected according to its purity.

Trypsin digestion and identification of trypsin digestion resistant proteins

Trypsin is commonly used in MS based proteomic approaches for its cleavage specificity C-terminal after lysine and arginine. Because cleavage efficiency impact on MS suitable sample yield and differential protein cleavage efficiencies would bias towards proteins that are more readily digested in proteomic approaches, we evaluated the trypsin proteolytic efficiency of our samples.

25µl of extracted protein samples were mixed with10 µl internal standard mix. Before addition of trypsin, 250µl NH_4HCO_3 and TCEP were added to give final concentrations of 50mM and 1 mM, respectively, and to lower the urea concentration to below 1M, compatible with trypsin activity. 10µl were extracted for SDS-PAGE control (control 3, Figure 5). 20 µg lyophilised modified trypsin (Promega) were resuspended in 50µl trypsin resuspension buffer (50 mM acetic acid). 10µl trypsin was added to each sample following incubation at 37°C for 4 h and at room temperature overnight. Digestions were stopped by adding formic acid to a final concentration of 1mM. 11µl of stopped reactions were taken for SDS-PAGE control (control 3, Figure 5).

First we estimated the trysin digestion efficiency by SDS-PAGE stained with SYPRO® Ruby Protein Gel Stain by side by side comparison of total lane fluorescence intensities, essentially as we estimated to protein extraction efficiencies (above) across all samples and found high yield (89.25%) and reproducibility (SE =/- 1.95%) (data not shown). We consistently observed two major bands with apparent molecular masses near 30kDa that were trypsin digestion resistant (Figure 5 arrows). The band migrating with an apparent molecular mass near 22kDa corresponds to the added trypsin (23.2k) that was not visible in control 3. In order to identify the trypsin resistant proteins in our samples, we blotted SDS-PAGE separated proteins onto a polyvinylidene difluoride membrane (PVDF, Sambrook and Russell) and determined the six N-terminal amino acids by Edman degradation [5]. Edman sequencing was carried out by AltaBioscience. The determined sequence for both protein bands was AEIYNK and uniquely matched to OmpF and PhoE only, by BLAST searches of the predicted K-12 (MG1655) proteome. The AEIYNK sequence is not located at the N-terminus but at amino acids 23-28 for OmpF and 22-27 for PhoE, respectively. OmpF and PhoE are outer membrane porins that are N-terminally processed by cleavage of the first 22 and 21 amino acid during translocation [6]. Unprocessed OmpF and PhoE have a molecular mass of 37.084 kDa and 36.835 kDa, respectively, and their observed mobility on SDS-PAGE agrees well will the processed proteins. We conclude that the major trypsin resistant proteins in *E.coli* NCM3722 are OmpF and PhoE, suggesting that our protocol produces proteome peptide samples, otherwise nearly quantitatively.

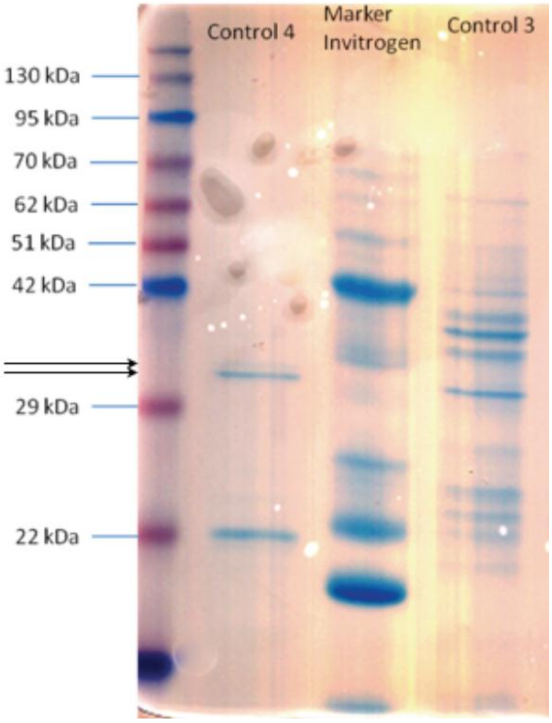

Figure 5: Trypsin digestion efficiency and trypsin resistant proteins OmpF and PhoE. Molecular weight standards are as indicated.

MRM Mass spectrometry (LC-MS/MS)

Mass Spectrometry (MS) –The trypsin digested samples were analysed on an Applied Biosystems QTrap MS coupled to an Agilent 1100 LC stack. The Agilent stack consisted of a Binary pump, Capillary pump, Well Plate autosampler and a column oven with integrated 6 port valve. The system was configured to load samples (50□l) onto a trap column (Agilent Zorbax SB 5□m x 0.3mm x 35mm) using the binary pump; the trap column was washed and then switched into the capillary flow; peptides were separated on a capillary column (Agilent SB 5 µm 0.5mm x 150mm column). The LC was interfaced to the MS with a Turbo Ion Spray Source. The loading/washing solvent was H_2O containing 0.2%COOH, 0.02%TFA at a flow rate of 150µl/min and the resolving solvent was a gradient system of 0% B to 40% B over 45 min at a flow rate of 10µl/min [(A)94.9% H_2O, 5% CH3CN, 0.1% COOH; (B) 94.9% CH3CN, 5% H_2O, 0.1% COOH]. The column oven was heated to $40^{o}C$; the valve was switched to direct the flow from the trap into the resolving column after a 5min wash. Typically the MS parameters were set to Curtain Gas 10psi, GS1 20psi, GS2 20psi, Interface heater on, TEM $150^{o}C$, DP 65, CE (collision energy) was set according to either empirically determined values or that estimated by MIDAS software (Applied Biosystems). The MS was used in "Trap" mode to acquire Enhanced Product Ion(EPI) scans for peptide sequencing and "Triple Quadruple" mode for Multiple Reaction Monitoring (MRM). Data analysis was performed using Analyst software (AB SCIEX).

Proteotypic peptides. Signature peptides for GS, IlvE, IDH, NtrC, and FNR were determined from trial MRM-MS runs of purified protein standards and from NCM3722 samples over-expressing these proteins from the plasmids provided from the appropriate ASKA(-) collection. The typical work flow to select the best signature peptides was to perform an *in silico* tryptic digest using MIDAS (AB SCIEX), the peptides were then analysed with ESPPredictor [7]. Additional *in silco* analysis looking at uniqueness, frequency of the peptides in the Global Proteome Machine (http://www.thegpm.org/) and tryptic cleavage probability maps [8] were also applied. Tryptic digests were analysed by targeting the top ranking peptides by Enhanced Product Ion (EPI) scans and also by Information Dependent Analysis (IDA) experiments . Daughter ion fragment information from the EPI's was used to derive Multiple Reaction Monitoring transition sets for the different peptides, typically 3-4 transitions per peptide were used; MS parameters were optimised and after testing against typical samples the two best transitions selected. A single proteotypic peptide for each protein was then selected for the final method (table 1)

Table 1: Overview of signature peptides and transitions for MRM-MS with PSAQ

**protein**	**peptide**	**internal standard (Y/N)**	**Q1**	**Q3**	**retention time**	**Collision energy**
GS	IPVVSSPK-1a	N	413.8	616.4	21.4	30
GS	IPVVSSPK-1b	N	413.8	517.3	21.4	30
GS	IPVVSSPK-1a-IS	Y	417.8	624.4	21.4	30
GS	IPVVSSPK-1b-IS	Y	417.8	525.3	21.4	30
IlvE	SVDGIQVGEGR-a	N	558.8	645.2	23.8	30
IlvE	SVDGIQVGEGR-b	N	558.8	930.3	23.8	30
IlvE	SVDGIQVGEGR-a-IS	Y	563.8	655.2	23.8	30
IlvE	SVDGIQVGEGR-b-IS	Y	563.8	940.3	23.8	30
IDH	GPLTTPVGGGIR-a	N	562.8	655.3	28.7	30
IDH	GPLTTPVGGGIR-b	N	562.8	857.5	28.7	30
IDH	GPLTTPVGGGIR-a-IS	Y	567.8	665.3	28.7	30
IDH	GPLTTPVGGGIR-a-IS	Y	567.8	867.5	28.7	30
NtrC	TLLTTALR-a	N	444.8	674.4	31.5	25
NtrC	TLLTTALR-b	N	444.8	561.3	31.5	25
NtrC	TLLTTALR-a-IS	Y	449.8	684.4	31.5	25
NtrC	TLLTTALR-b-IS	Y	449.8	571.3	31.5	25
FNR	LAAFIYNLSR-a	N	584.3	652.3	38.1	31
FNR	LAAFIYNLSR-b	N	584.3	765.4	38.1	31
FNR	LAAFIYNLSR-a-IS	Y	589.4	662.3	38.1	31
FNR	LAAFIYNLSR-b-IS	Y	589.4	775.4	38.1	31

References

1. P. Picotti and R. Aebersold, *Nat Methods*, 2012, **9**, 555-566.
2. J. L. Lopez, *Journal of chromatography. B, Analytical technologies in the biomedical and life sciences*, 2007, **849**, 190-202.
3. G. Picard, D. Lebert, M. Louwagie, A. Adrait, C. Huillet, F. Vandenesch, C. Bruley, J. Garin, M. Jaquinod and V. Brun, *Journal of mass spectrometry : JMS*, 2012, **47**, 1353-1363.
4. B. Volkmer and M. Heinemann, *PLoS One*, 2011, **6**, e23126.
5. P. Edman, *Archives of biochemistry*, 1949, **22**, 475.
6. S. O. Hagge, H. de Cock, T. Gutsmann, F. Beckers, U. Seydel and A. Wiese, *J Biol Chem*, 2002, **277**, 34247-34253.
7. V. A. Fusaro, D. R. Mani, J. P. Mesirov and S. A. Carr, *Nature biotechnology*, 2009, **27**, 190-198.
8. H. C. Gasteiger E., Gattiker A., Duvaud S., Wilkins M.R., Appel R.D., Bairoch A. , *Protein Identification and Analysis Tools on the ExPASy Server;*, Humana Press, 2005.
